# Treating recurrent cases of squamous cell carcinoma with radiotherapy

**DOI:** 10.3747/co.v15i5.196

**Published:** 2008-10

**Authors:** J. Wong, D. Breen, J. Balogh, G.J. Czarnota, J. Kamra, E.A. Barnes

**Affiliations:** * Department of Radiation Oncology, University of Toronto, Faculty of Medicine, Odette Cancer Centre, Sunnybrook Health Sciences Centre, Toronto, ON; † Department of Medical Sciences, Faculty of Medicine, University of Toronto, Toronto, ON

**Keywords:** Squamous cell carcinoma, chronic lymphocytic leukemia, radiotherapy

## Abstract

Patients with chronic lymphocytic leukemia (cll) are at a significantly increased risk of developing cutaneous squamous cell carcinoma (scc), in part because of their impaired immunosurveillance. Here, we report the cases of 4 patients with cll who had locally aggressive cutaneous scc managed with radiotherapy for local recurrence following surgical excision. All tumours were located in the head-and-neck region. All patients initially achieved complete regression of disease; however, 2 had local recurrence a mean of 8 months after treatment completion. One patient died from progressive scc. Our findings agree with the high rates reported in literature of multiple tumours, local recurrence, metastases, and mortality from scc in patients with cll. Radiotherapy plays an important role in patient management, and it is the recommended treatment modality when complete surgical excision of disease would result in anatomic and functional defects. Radiotherapy is often used in the case of local recurrence after one or more attempts at surgical excision. Dose escalation through intensity-modulated radiotherapy, hyperfractionation, or novel treatment techniques such as high-intensity focused ultrasound may be explored to improve local control of scc lesions. To optimize patient outcomes, cutaneous scc arising in patients with a history of cll should be managed and followed in a multidisciplinary clinic, with regular skin surveillance and prompt treatment.

## 1.INTRODUCTION

Chronic lymphocytic leukemia (cll) is the most common adult leukemia in the United States and Western Europe^1^. Chronic lymphocytic leukemia is a clonal disorder characterized by an increased number of mature B lymphocytes with prolonged survival. This incurable disease can have either an indolent or an aggressive course; median survival is 7 years (range: a few months to several decades). Death is typically the result of infection or secondary malignancy^2^,^3^.

Patients with cll are at an increased risk of developing secondary malignancies, including cutaneous )squamous cell carcinoma (scc 4–6. The increased incidence of cutaneous scc is thought to be a result of the impaired immunosurveillance seen in cll patients, together with a lack of repair of accumulated damage from ultraviolet radiation on sun-exposed skin. In immunocompetent patients, high rates of local control and cure of cutaneous scc are achieved through electro-desiccation and curettage, surgical excision, or local radiotherapy^7^. However, in patients with cll, high rates of multiple tumours, local recurrence, metastases, and mortality from cutaneous scc are seen^8^–^13^.

Several reports in the literature have described the role of surgical excision, including Mohs micrographic surgery, in the treatment of cutaneous scc in cll patients^12^,^13^. However, the role of radiotherapy has not been previously described. Given the high rate of local recurrence after primary surgery, the multifocal nature of the disease, and the rapid rate of tumour growth, radiotherapy plays an important role in the management of cutaneous scc in this patient population. Here, we report on 4 patients with cll and locally aggressive cutaneous scc that were managed at our center with radiotherapy after local recurrence following surgical excision.

## 2.CASE SELECTION

The multidisciplinary non-melanoma skin cancer clinic at the Odette Cancer Center includes a radiation oncologist, a plastic surgeon, and a dermatopathologist. These practitioners assess patients and jointly render a treatment recommendation. A patient database was started in June 2005 to record patient demographics, tumour characteristics, and treatment-related information. We searched the database to identify patients with a history of cll who were treated with radiotherapy after local recurrence following surgical excision of a cutaneous scc. Patient information including age, sex, and time since cll diagnosis were recorded. The patients’ cll treatment histories were recorded. Information on treatment-related factors, including scc location, number of excisions, final surgical pathology, time to recurrence from last surgery, tumour size at the time of radiation, radiation treatment details, and treatment outcomes were also recorded. For patients having more than 1 recurrent scc treated with radiation, the treatment history of only the first scc was recorded for the purposes of this study. The total number of scc lesions (including local recurrences) treated with surgery or radiotherapy were also documented.

## 3.RESULTS

We identified 4 patients with a history of cll who were treated with radiotherapy for a cutaneous scc after local recurrence following surgical excision from June 2005 to August 2007. Tables I and II show patient demographics and information on the tumour and on treatment characteristics. Mean age of the patients was 84 years, and all scc tumours were located in the head-and-neck region. All patients had undergone 1 or 2 surgical excisions before radiation delivery, and the final surgical pathology had shown positive margins for invasive disease in 2 of the 4 patients. Time to recurrence was much shorter in patients with positive margins than in those with negative margins: a mean of 2.5 weeks as compared with 21 weeks. All patients developed metachronous cutaneous scc tumours (separate lesions developing over time). Total scc lesions (including local recurrences) treated with surgical excision or radiation numbered 5, 10, 11, and 4 for patients 1, 2, 3, and 4 respectively.

Radiotherapy was delivered with curative intent for all patients. The 2 patients who had positive margins after excision had multiple nodules, which recurred rapidly (4 or fewer weeks) in the surgical bed. In those patients, the scc lesions continued to grow during the course of radiation; the radiation treatment field had to be enlarged to encompass the disease.

**FIGURE 1:**
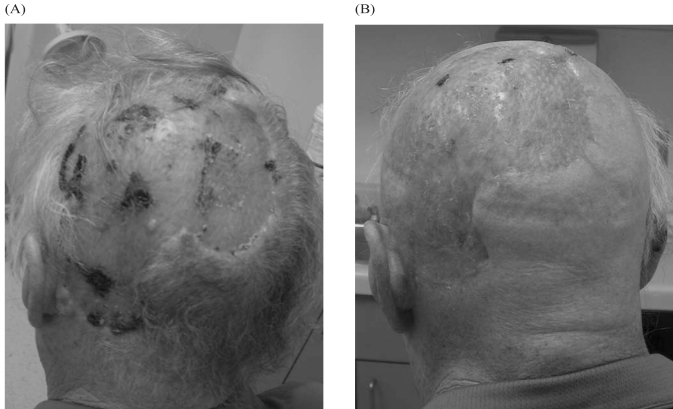
Case 4. (A) Pre- and (B) 2-month post-treatment images, showing complete clinical response to radiation treatment.

Three patients were prescribed a dose of 50 Gy in 20 fractions. One patient, case 4, was treated twice daily (40 Gy in 20 fractions) because of rapid tumour growth. The result was dramatic tumour regression at 1 month, and no visible evidence of disease at 2 months’ follow-up after radiation treatment (Figure 1). However, at the time of this patient’s 2-month visit, a new 1.0-cm scc was detected in the region of the right temple. Because the patient was being followed in the multidisciplinary clinic, a plastic surgeon was able to examine the patient and excise the lesion that same day.

Table III presents patient outcomes. Two patients achieved complete regression of disease; 2 had local recurrence at 5 months and 11 months after treatment completion. Longer follow-up is required for the other 2 patients to determine whether they will have a durable response to radiation.

Table IV shows cll history in the patients, including chemotherapy use. One patient received chemotherapy, including fludarabine, while receiving treatment for scc. This patient ultimately died from progressive scc.

## 4.DISCUSSION

The present report is the first to highlight the important role of radiation therapy in the management of patients with aggressive cutaneous scc and a history of cll. We describe 4 patients seen in our multidisciplinary non-melanoma skin cancer clinic who had local recurrence following surgical excision of a cutaneous scc in the head-and-neck region, where further surgery was not recommended, and the treatment modality of choice was local radiotherapy. Although the final outcome for all patients was not total eradication of local disease, radiation was able to provide local control for several months.

Adami *et al*^6^ previously reported that patients with cll have a relative risk of developing cutaneous scc that is 8.6 times the risk seen in the general population, with a progressive increase after the first decade of cll diagnosis. The increased incidence and aggressive behaviour of cutaneous scc is thought to be related to the inherent immune defects seen with cll (some of which occur as a consequence of cll therapy), together with dna damage from ultraviolet radiation. Ultraviolet radiation generates specific mutations (thymidine dimers) in the *TP53* tumour-suppressor gene. Keratinocytes with *TP53* mutations cannot undergo apoptosis and instead undergo clonal expansion, which manifests clinically as actinic keratosis. Uncontrolled proliferation of abnormal cells leads to squamous-cell carcinoma *in situ* and invasive squamous-cell carcinoma 7.

Chronic lymphocytic leukemia is characterized by an accumulation of immunologically incompetent B cells. These cells produce reduced amounts of normal immunoglobulins in response to antigenic stimuli. Abnormalities of the normal B, T, and natural killer cell functions are also seen^14^. The chemotherapeutic agents used to treat cll also cause immunosuppression. Fludarabine in particular causes depletion of T lymphocytes for many months, and because the T-cell response plays a significant role in controlling scc, fludarabine use may cause scc to flare or metastasize rapidly. Previous case reports have shown rapid development and progression of scc after fludarabine use^15^,^16^. One patient in our series with multiple scc lesions had been on fludarabine for a month before the rapid growth of her scc, and she ultimately died of progressive scc.

Our series agrees with other work reporting high rates of multiple tumours, local recurrence, metastases, and mortality from cutaneous scc in patients with cll 8–13. In the series from Weimar *et al.*^8^, all the patients developed tumour recurrence and regional metastasis after various treatments. Perez–Reyes and Farhi 9 found a 50% rate of regional metastasis, a 37% tumour multiplicity rate, and a 33% rate of tumour persistence or recurrence following various treatments in patients with cll and scc of the head and neck.

Ascertaining clinically whether palpable head-and-neck nodes are the result of infiltration by cll or scc is difficult, and because nodal pathology was not obtained in all patients in our series, we cannot comment on the rate of regional metastases. In fact, both types of metastases may be present, and cancer-to-cancer metastases of cutaneous scc to a lymph node replaced by cll has been reported^17^.

In our series, all patients had multiple tumours, highlighting the aggressive behaviour of scc in patients with cll. All patients responded to radiotherapy with shrinkage and eradication of disease based on clinical exam. However, the duration of response was limited, because 2 patients recurred (one at 5 months, the other at 11 months). Longer follow-up is required for the other 2 patients, to assess whether they will have a durable response to radiation.

Radiotherapy is the recommended treatment modality for the radical primary treatment of non-melanoma skin cancer when the anatomic, functional, and cosmetic outcome would be better than that with surgery, with an equivalent cure rate; when the tumour is not amenable to complete surgical resection because of size or location; when patient preference dictates; or when contraindications to surgery exist. Examples include small lesions on the lip, eyelid, or nose (which are difficult to reconstruct surgically), and larger lesions on the scalp, ears, and forehead. Radiation is often used when tumours have recurred after surgery, especially when re-excision would cause functional or cosmetic defects or would be expected to result in positive margins, or when, in the adjuvant setting, the risk of local recurrence is perceived to be high.

For the patients in our series, further surgery was not recommended because of the site and extent of recurrence. Large radiation fields were needed to encompass the disease and the surgical bed at risk for microscopic disease. When metachronous lesions or regional metastases develop in the head-and-neck region, repeat courses of radiation can be challenging, because the treatment fields have the potential to overlap. Intensity-modulated radiation therapy (imrt), wherein radiation can be conformally delivered to the target volume while minimizing dose to nearby normal or previously treated tissue, may be an option in some circumstances.

When scc lesions recur or when residual disease remains after radical radiation, management options are limited. Further radiation is rarely possible because of the previous treatment having approached the tolerance doses of normal tissue within the treated field. Surgery may not be possible because surgical excision was not recommended initially, unless the recurrent tumour was small, localized, and located in a place where excision and reconstruction would not cause significant morbidity. Furthermore, operating in a previously irradiated field may be suboptimal because of radiation fibrosis and compromised vasculature. The high rate of local relapse combined with rapid tumour growth makes a reasonable case for exploration of the role of hyperfractionation and dose escalation, or of alternative treatment modalities such as high-intensity focused ultrasound.

## 5.CONCLUSIONS

The management of cutaneous scc in patients with cll is challenging. Patients are typically elderly, with multiple comorbidities. Patient care is ideally managed in a multidisciplinary setting with involvement of a dermatologist, plastic surgeon, radiation oncologist, and hematologist. Multiple lesions often develop over time, and therefore regular skin surveillance is recommended. Questionable skin lesions should be biopsied early and should be completely excised if malignant. A low rate of local control, rapid tumour growth, and a high rate of metastasis are the causes of death in many of these patients. Early detection and aggressive treatment are vital for reducing morbidity and mortality. Recurrent lesions can be effectively treated with local radiotherapy. Dose escalation through imrt or hyperfractionation to improve local control is worthy of exploration. Systemic therapy to reduce the risk of metachronous lesions and regional and distant metastases should also be considered in selected patients.

## Figures and Tables

**TABLE I:** Patient characteristics and tumour characteristics for the site treated

Case	Age	Sex	scc site	Excisions before rt (n)	Final surgical pathology (margins)	Time to local recurrence from last surgery (weeks)
1	92	Male	Temple	1	Positive	1
2	80	Female	Cheek	1	Negative	22
3	82	Female	Forehead	2	Negative	20
4	79	Male	Scalp	2	Positive	4

scc = squamous-cell carcinoma; rt = radiation therapy.

**TABLE II:** Radiation treatment details

Case	Tumour size at time of radiation treatment	Radiation field size (cm)	Treatment prescription [dose (Gy)/fractions]	Field enlarged during treatment?
1	Numerous small nodules in surgical bed (largest, 1.0 cm)	13.0 × 14.0	50/20	Yes
2	4.0 × 3.0 cm	10.0 × 10.0	50/20	No
3	2 Nodules in surgical bed (largest, 3.5 × 1.5 cm)	10.0 × 10.0	50/20	No
4	Numerous nodules in surgical bed (largest, 3.0 × 2.0 cm)	14.0 × 21.0	40/20 (twice daily)	Yes

**TABLE III:** Patient outcomes

Case	Local outcome	Metachronous lesions?	Current status
1	Local control at 2 months	Y	Alive
2	Local recurrence at 11 months	Y	Alive
3	Local recurrence at 5 months	Y	Deceased (from scc)
4	Local control at 2 months	Y	Alive

**TABLE IV:** History and treatment of chronic lymphocytic leukemia (cll)

Case	Time since cll diagnosis (years)	Chemotherapy agents since cll diagnosis	Chemotherapy used during/after rt?
1	5	No	No
2	18	Chlorambucil	No
3	4	Chlorambucil, fludarabine	Yes (both agents used)
4	8	Chlorambucil	Yes
